# Changes in objectively measured lifestyle factors during the COVID-19 pandemic in community-dwelling older adults

**DOI:** 10.1186/s12877-022-03043-1

**Published:** 2022-04-14

**Authors:** Takuya Ataka, Noriyuki Kimura, Atsuko Eguchi, Etsuro Matsubara

**Affiliations:** Department of Neurology, Faculty of Medicine, Oita University, Oita, Japan

**Keywords:** Aged, COVID-19, Japan, Lifestyle, Wearable sensor

## Abstract

**Background:**

In this manuscript, we investigate whether objectively measured lifestyle factors, including walking steps, sedentary time, amount of unforced physical activity, level of slight and energetic physical activity, conversation time, and sleep parameters, were altered before and during the COVID-19 pandemic among community-dwelling older adults.

**Methods:**

Data were obtained from a prospective cohort study conducted from 2015 to 2019 and a subsequent dementia prevention study undertaken in September 2020. Community-dwelling adults aged ≥ 65 years wore wearable sensors before and during the pandemic.

**Results:**

A total of 56 adults were enrolled in this study. The mean age was 74.2 ± 3.9 years, and 58.9% (*n* = 33) of the participants were female. Moderate and vigorous physical activity time significantly decreased, and sedentary time significantly increased during the pandemic.

**Conclusions:**

This is the first study to demonstrate differences in objectively assessed lifestyle factors before and during the COVID-19 pandemic among community-dwelling older adults. The findings show that the pandemic has adversely affected physical activity among older adults living on their own in Japan.

## Background

The coronavirus (COVID-19) pandemic is threatening the health of human populations globally. Following the emergence of COVID-19 in late 2019, countries experienced the first to sixth waves of the disease. As of March 3, 2022, there have been over 430 million cases of the disease and over 5 million deaths worldwide [1]. The COVID-19 pandemic has forced governments to impose strict confinement rules on their citizens. In Japan, more than 5 million cases of COVID-19 have been confirmed, including approximately 24,000 deaths [2]. The government declared a state of emergency as the first wave hit, which was not legally binding but prompted many people to wear face masks, practice social distancing, and refrain from going out. The declaration, released on April 16, 2020, applied to the entire country and was in place for more than a month. People restricted their daily activities to prevent the spread of COVID-19 while the rules were in place. These control measures potentially resulted in physical inactivity and social isolation leading to increased levels of psychological stress [3]. Several studies have examined changes in lifestyle factors among young adults that occurred during the period of confinement. The results showed that COVID-19 restrictions had adverse effects on physical activity and sleep in younger adults [4, 5]. However, few studies have demonstrated whether the COVID-19 pandemic affected the lifestyles of older adults living alone in the community. Physical inactivity, sleep disturbances, and social isolation are major problems in older adults with increasing life prospects [6–9] and are associated with reduced well-being and an increased risk of mental impairment, major preexisting conditions, and death in older adults [10–15]. Thus, comparing lifestyle factors before and during the pandemic is important in understanding their indirect adverse impacts on physical health among older adults. Previously, to examine the risks and protections associated with lifestyle factors in relation to dementia, we conducted a prospective cohort study that objectively measured lifestyle factors in community-dwelling older adults using wearable sensors from August 2015 to March 2019 [16]. Moreover, a subsequent dementia prevention study provided us with an opportunity to measure lifestyle factors objectively in a subgroup of these participants during the pandemic. These noninvasive and cost-effective wearable sensors were used to objectively assess total daily effort and sleep without recall bias, particularly in older adults. Herein, we aimed to evaluate the changes that occurred in objectively measured lifestyle factors before and during the COVID-19 pandemic in community-living older adults.

## Methods

### Study design and participants

A total of 56 participants (33 females, mean age 74.2 ± 3.9 years, years of education 11.7 ± 2.0 years) were enrolled among a population of community-dwelling older adults living in Usuki, a rural city in Oita Prefecture, Japan. The population included both those who live alone and those who do not. In the present study, the inclusion criteria met by all participants were as follows: 1) aged 65 and over, 2) lived in Usuki, 3) physically and psychologically healthy, 4) no dementia, and 5) conducted daily living activities independently. All subjects were instructed to wear wristbands (Silmee™ W20, TDK Corporation Tokyo, Japan) throughout the day, except when showering, for a week during September 2020. These subjects had previously participated in a prospective cohort study with a continuous follow-up that analyzed the association between objectively quantified lifestyle factors and mental function from August 2015 to March 2019. In this study, we defined lifestyle factors as physical movement, sleep habits, and social interaction. During that cohort study, we assessed lifestyle factors using wearable sensors every 3 months for 3 years and calculated average annual data [16]. Overall, the average annual data for 2016 and 2018 and the average weekly data for 2020 were collected for analysis.

### Data obtained by the wearable sensors

For movement detection, we used a tri-axis accelerometer that measures acceleration in three perpendicular axes. Accelerometers are based on the principle of differential capacitance, which results from the movement of the sensing element due to acceleration. Data were recorded continuously and analyzed every minute. We defined nonwearing time as the time when heart rate counts were zero. We analyzed the data for participants who had at least three days of valid accelerometer monitoring during the measurement period and at least three hours of valid accelerometer monitoring per day. We used our wearable sensors to assess various lifestyle parameters, including moving steps, metabolic equivalents of tasks (METs), total sleep time (TST), sleep effectiveness, wake time after sleep onset (WASO), number of awakenings, and conversation time. We summarized the measured values of these parameters into the sum of the sensor data collected on each day and their average values over the entire length of the period. Each parameter was represented as an average daily value. Before our study, we validated the accuracy of our measurements of hiking steps, talking time, and sleep time by comparing them with data from video observations of healthy older subjects.

### Physical activity

We defined walking steps as a movement in the frequency range of 2–3 Hz of acceleration detected by the sensor. The device computed the intensity of activities as METs using algorithms developed by the product designer. Sedentary behavior was defined as activities that involved ≤ 1.5 METs such as sitting, lying down, or watching television. Light physical activity (LPA) was defined as activities that involved 1.5–3.0 METs such as slow walking, laundry, cooking food, washing dishes, or vacuuming, whereas moderate and vigorous physical activities (MVPA) were defined as activities that involved ≥ 3 METs such as walking, jogging, or ascending and descending stairs.

### Sleep

We evaluated sleep–wake parameters using the magnitude of acceleration and cumulative energy synthesized by a triaxial accelerometer. The data were verified and corrected visually by a qualified technician. Bedtime was determined according to the number of activities logged by the wristband sensor. Sleep factors, including TST, WASO, and sleep efficiency, and the awakening count were measured between 6:00 pm and 5:59 am (the following day). The time of sleep onset was defined as the time when the resting state began, with no movement for more than 20 min. We evaluated sleep fragmentation by using WASO, sleep effectiveness, and awakening counts. We defined nightly awakenings as 5 to 90 min of continuous movement during a continuous sleep period. Sleep effectiveness was determined as the percentage of TST over bedtime. In this study, sleep diaries were not utilized, rather, the total bedtime was measured using TST and WASO.

### Conversation time

This sensor was able to detect whether an adult or someone nearby had made utterances. The sensor does not record the sound itself but rather the sound as sound pressure and duration. Although we were not able to exclude the speech of someone nearby from the audio data or self-chat, the contribution of the participant in the conversation itself was judged to be a valuable sign of social activity. The microphone on the wearable sensor cannot detect the substance of the chat, but it can collect data as sound every minute. We analyzed sound data to evaluate the duration of the conversation. Our wearable sensor can detect the sound pressure level of utterances within a 2 m radius of the device. The vibration level, considered utterances at this distance, was 55–75 dBA. Furthermore, the incidence band corresponding to the human voice was extracted as a signal frame from the sound data within the vibration range. A chat was defined as a period of one minute during which there were more than four sound frames.

### Statistical analysis

The annual average data for 2016 and 2018 and the weekly average data for 2020 before and during the pandemic, respectively, were used for statistical analyses. A repeated-measures analysis of variance (rANOVA) was conducted to compare nine variables of lifestyle factors (walking steps, sedentary time, LPA, MVPA, chat time, TST, WASO, sleep effectiveness, and waking time count) before and during the pandemic. All statistical analyses were conducted using SPSS statistical software (version 25.0, IBM Corporation, USA) and Prism (version 7.00, GraphPad), and all *p value*s of < 0.05 were considered statistically significant.

## Results

**Fig. 1:**
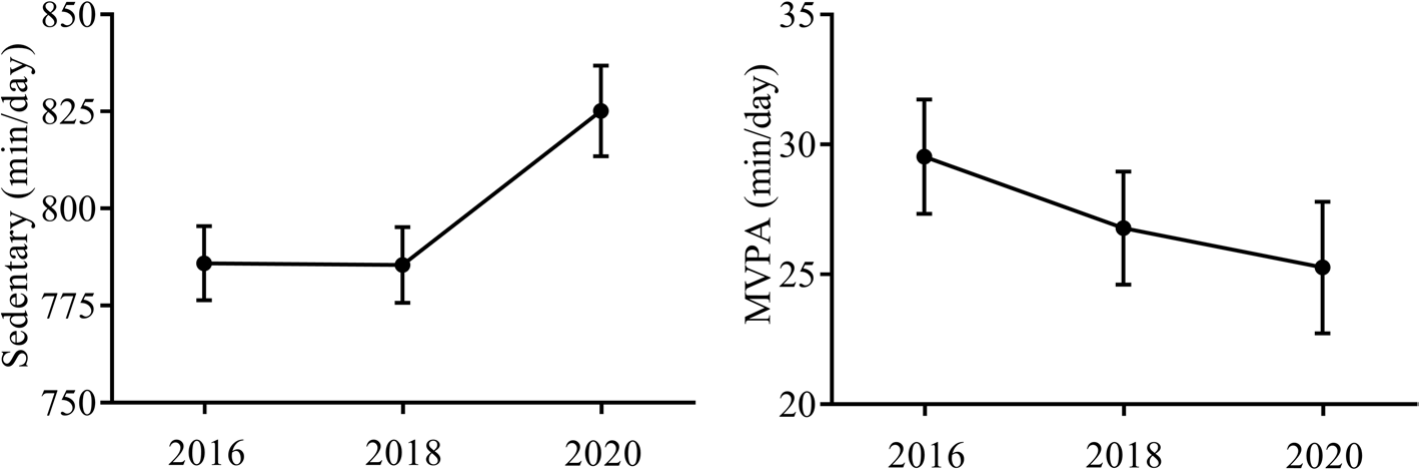
Temporal variations in the objectively measured MVPA and inactive time. Sedentary time significantly increased during the pandemic compared with that before the pandemic. The time spent undertaking MVPA drastically diminished. (*p* < 0.05*). Abbreviations: *MVPA* moderate-vigorous physical activity.

The demographic and clinical characteristics of all participants are summarized in Table 1. The mean age was 74.2 ± (3.9) years, and 41.1% were male (*n* = 23). The mean education level was 11.7 ± 2.0 years, and the mean Mini-Mental State Examination score was 27.4 ± 2.6 points. The temporal changes in the objectively measured lifestyle factors are shown in Table 2 and Fig. 1. The amount of daily sedentary time increased (*p* = 0.028), and the daily MVPA time significantly decreased (*p* = 0.042). No significant changes were noticed in daily walking steps, chat time, LPA time, or other sleep parameters.

**Table 1:** Clinical and demographical characteristics of participants

Characteristics	
Age, mean (SD), years	74.2 (3.9)
Sex	
Male, n (%)	23 (41.1%)
Female, n (%)	33 (58.9%)
Education level, mean (SD), years	11.7 (2.0)
MMSE, mean (SD)	27.4 (2.6)
Medical history	
Hypertension, n (%)	30 (53.6%)
Diabetes, n (%)	13 (23.2%)
Hyperchoresterolemia, n (%)	20 (35.7%)

*MMSE* Mini-Mental State Examination

**Table 2:** Temporal changes in objectively measured lifestyle factors

				Repeated measures ANOVA
	2016	2018	2020	*p* value
Walking steps, mean (SD), steps/day	5727.3 (2506.0)	5449.6 (2547.4)	4985.3 (3092.8)	0.066
Talking ratio, mean (SD), min/day	217.2 (77.8)	222.0 (70.0)	217.1 (78.5)	0.19
TST, mean (SD), min/day	403.1 (66.7)	406.9 (86.2)	400.8 (89.1)	0.67
WASO, mean (SD), min/day	16.4 (9.3)	16.9 (9.6)	17.0 (13.1)	0.44
Sleep efficiency, mean (SD), %/day	96.1 (2.2)	95.9 (2.5)	95.7 (3.4)	0.49
Awake time count, mean (SD), counts/day	0.44 (0.28)	0.43 (0.28)	0.45 (0.33)	0.51
Sedentary time, mean (SD), min/day	785.3 (69.7)	787.2 (72.2)	829.1 (87.4)	0.028
LPA, mean (SD), min/day	26.9 (18.3)	25.5 (15.1)	23.9 (14.4)	0.80
MVPA, mean (SD), min/day	29.4 (16)	26.5 (16)	24.8 (18.5)	0.043

*LPA* light physical activity, *MVPA* moderate-vigorous physical activity, *TST* total sleep time, *WASO* time awake after sleep

## Discussion

We investigated the changes in lifestyle factors before and during the COVID-19 pandemic among community-dwelling older adults using wearable sensors. Although multiple reports have assessed the changes in physical activity or sleep parameters individually during the pandemic among younger adults, to the best of our knowledge, this is the first study to investigate the changes in objectively measured lifestyle factors during the pandemic in older adults. Our results provide novel and interesting insights that will help in developing strategies to prevent the indirect health impacts of the pandemic on older adults. Sedentary time significantly increased, while daily MVPA time decreased. The present study has several strengths, including the objective measurement of various lifestyle factors among community-dwelling older adults and the comparison of those measurements obtained before and during the pandemic.

The truly interesting finding of the present study was that the extent of sedentary time was significantly greater during the pandemic than before the pandemic. Few studies have compared the intensity of exercise performed by people before and during the pandemic. One study showed that the time spent undertaking MVPA decreased during a semilockdown period compared with that measured before the pandemic in Chinese young adults [17]. Similarly, our results showed that the daily time spent undertaking MVPA decreased significantly during the pandemic compared with that measured before the pandemic; however, the time spent on MVPA had already decreased between 2016 and 2018. Thus, we were unable to determine whether the decreased amount of MVPA was caused by aging or the pandemic. Physical inactivity results in increased risks of developing metabolic diseases [18], cardiovascular diseases [19], and immune dysfunctions [20]. For older adults, even short-term physical inactivity can result in muscle loss or frailty [3]. Muscle loss has a negative relationship with metabolic syndromes and cognitive function. The World Health Organization (WHO) has recommended regular physical activity during the pandemic for people of all ages to maintain their health and well-being [21]. Our results suggest that the pandemic indirectly had an adverse impact on physical activity among older adults and support the recommendations of the WHO. Moreover, our results indicated that there were no changes in sleep parameters according to the measurements of older adults taken before and during the pandemic. Previous studies that objectively measured sleep time using smartphones showed that sleep time significantly increased during the pandemic compared with that before the pandemic among young adults [4, 22, 23]. Possible explanations for this discrepancy may have been the differences in the ages of the participants or the severity of psychological stress during the pandemic. The psychological stress endured by Japanese people was milder than that endured by people from other countries because the lockdown in Japan was not as strict.

This study showed no change in conversation time during the pandemic. Few studies have examined the association between conversation time and the pandemic. In this study, we used conversation time as a surrogate marker of social interaction. Conversation potentially includes talking to others, including family members, talking to oneself, and the sound of TV or radio in our method. The decrease in the number of walking steps suggested a possible decrease in interaction with others; however, the conversation time did not actually decrease. It is possible that the time talking to others decreased, while the time spent self-chatting or watching the radio or TV at home increased, but but we cannot reveal the detail in this study.

To improve the adverse impacts on physical activity in older people, we recommend the activities suggested by the WHO, such as engaging in exercise online, dancing to music, strength and balance training, and climbing stairs [21]. In addition, we recommend breaking up and standing up regularly while sitting. There were several limitations to the present study that should be considered. First, noise from the television or radio might be detected during the conversation using the chosen detection method, which was based on vibrations and frequencies. In addition, sleep may have been incorrectly categorized as chat time during television broadcasting or listening to the radio. However, a previous study showed that only 6.4% of sleeping time was included in the daily chat. Second, a small number of participants were included in the present study. During a pandemic, it is very difficult to study large populations, but lifestyle factors need to be evaluated in larger populations while maintaining infection control in the future.In summary, this study is the first to confirm the indirect and adverse effects of the COVID-19 pandemic on physical health among community-dwelling older adults. A decline in the amount of daily MVPA time and an increase in sedentary time were observed during the pandemic compared with before the pandemic. These results will contribute to our knowledge about the reduction of physical inactivity during the pandemic in older adults.

## Data Availability

The datasets used during the current study are available from the corresponding author upon reasonable request.
